# Effect of Geometric Design on the Mechanical Performance of Digital Light Processing (DLP)-Printed Microneedles

**DOI:** 10.3390/mi16111221

**Published:** 2025-10-27

**Authors:** Tuba Bedir, Siba Sundar Sahoo, Sachin Kadian, Oguzhan Gunduz, Roger Narayan

**Affiliations:** 1 Center for Nanotechnology and Biomaterials Application and Research (NBUAM), Marmara University, 34854 Istanbul, Turkey; 2 Department of Materials Science and Engineering, North Carolina State University, Raleigh, NC 27695, USA; 3 Joint Department of Biomedical Engineering, University of North Carolina, Chapel Hill, NC 27599, USA; 4 Department of Metallurgical and Materials Engineering, Faculty of Technology, Marmara University, 34854 Istanbul, Turkey

**Keywords:** microneedles, 3D printing, digital light processing, geometric optimization, mechanical stability

## Abstract

This study describes the processing of microneedle (MN) arrays with three different heights of arrowhead (600 µm (A1), 800 µm (A2), and 1000 µm (A3)), pyramid (600 µm (P1), 800 µm (P2), and 1000 µm (P3)), and turret (600 µm (T1), 800 µm (T2), and 1000 µm (T3)) designs using a digital light processing (DLP)-based 3D printing method. The 3D-printed MNs were examined for their morphological characteristics and mechanical performance. Scanning electron microscopy (SEM) imaging confirmed that all of the MNs were fabricated without fracture or bending. Each design exhibited distinct structural characteristics: arrowhead MNs displayed a well-defined morphology with sharp tips, pyramid MNs showed slight layering, and turret MNs, characterized by a wider base and sharp tips, had a smoother surface compared to the other designs. Mechanical tests revealed that the arrowhead MNs carried less load and were more prone to bending, while the pyramid and turret designs provided higher mechanical stability and penetration capacity. The pyramid design (P3) showed the highest mechanical strength, while turret MNs offered a more stable performance despite lower penetration capacity. These findings highlight the critical role of geometric design in optimizing MN performance for effective transdermal drug delivery.

## 1. Introduction

A promising strategy for delivering medications through the skin barrier involves the use of microneedle (MN) patches, which are arrays of microscale projections. These patches enable minimally invasive drug delivery by generating microchannels in the epidermis, facilitating the diffusion of therapeutic agents across the stratum corneum layer of the epidermis [1,2]. In comparison to conventional hypodermic needles, MNs provide a more comfortable and less invasive alternative. Due to their microscale structure, MNs bypass the pain-sensitive nerve endings in the skin, making them an appealing option, especially for individuals with needle phobia [2]. MNs have proven effective in administering diverse therapeutic compounds, including small molecules, nanoparticles, microparticles, and vaccines, into the epidermal and dermal layers [3]. A variety of materials, such as glass, silicon, metals, polymers, and ceramics, have been utilized in the fabrication of MNs. However, concerns remain regarding the biocompatibility and restricted drug loading capacity of MNs, which pose challenges to their safe and effective use [4,5].

Various parameters influence the design and performance of MNs. To ensure successful skin insertion across various MN materials, geometric features, and array configurations, it is important to enhance the understanding of the effect of MN design on skin interaction [6]. Key factors such as material composition (e.g., silicon, polymers, or metals), spatial arrangement (e.g., triangle, square, hexagon, or radial), and geometric attributes (e.g., tip diameter, base diameter, center-to-center spacing, and base-to-tip ratio) significantly affect penetration depth [7]. To comply with the Food and Drug Administration (FDA) requirements for MN-based medical devices, it is essential to optimize parameters such as MN length, sharpness, arrangement, and puncture rate [7,8]. Recent studies have highlighted the need for greater control over these MN design parameters [9,10,11]. The purpose of achieving this level of control is to ensure both the mechanical reliability of the MNs during insertion and the therapeutic effectiveness of the drug delivery functionality of the MNs. Since an effective MN must penetrate the skin without fracture, both the insertion force and the failure strength of the MN material are critical considerations [12]. For instance, pyramidal MNs demonstrate better mechanical strength than conical ones because they exhibit a larger cross-sectional area at the same base width [13,14]. Additionally, increasing the base width while reducing the aspect ratio further enhances the structural integrity of pyramidal MNs [14]. Similarly, a shorter tip length combined with a wider base width increases the failure force, improving overall mechanical stability [15]. Moreover, the MN design plays an important role in determining the quantity of therapeutic agent that can be effectively delivered by the MN. Due to their small size, delivering clinically relevant drug dosages using MNs is challenging, with the exception of highly potent compounds [16]. As a result, clinical trials have primarily focused on MN delivery of vaccine and hormone products, which require only microgram quantities to achieve clinical efficacy [17]. Increasing the MN height expands the drug-loading capacity by increasing the volume; however, increasing the MN height may be associated with a concomitant increase in patient discomfort. Likewise, adding more MNs to an array allows for a greater amount of drug to be delivered but also increases the insertion force that is required. Thus, exercising precise control over the MN design parameters is essential for balancing mechanical robustness, drug loading efficiency, and patient comfort, facilitating the development of clinically viable MN technologies [9].

More recently, additive manufacturing (AM), or 3D printing, has garnered attention as a promising approach for fabricating MN arrays, either directly or through the production of master molds for replication of MN arrays [18]. This technology provides exceptional control over MN design, enabling precise control over size, shape, and structural features [19,20,21]. AM has facilitated the straightforward fabrication of intricately designed MNs, such as arrowhead shapes, angled structures, and honeybee-inspired forms, which are often difficult to produce using traditional fabrication techniques [2,22]. Vat photopolymerization-based AM techniques, which involve selectively curing a liquid photopolymer using light from a lamp or a laser source, provide sufficient resolution for the fabrication of MNs with small-scale features [23]. Several types of vat photopolymerization-based AM techniques, such as two-photon polymerization [24,25] and continuous liquid interface production [9], have been used to prepare MNs. However, these techniques are expensive and are not widely accessible [2]. To address this, several more affordable vat photopolymerization-based AM methods, including digital light processing (DLP), have been investigated for fabricating MN arrays. In digital light processing, a digital mirror device projects light from a lamp source onto a layer of liquid photopolymer, which results in the solidification of the photopolymer. This light exposure process is repeated to manufacture a solid structure in a layer-by-layer manner [18]. Unlike the use of a laser for vat photopolymerization-based AM, which exposes each layer to light in a point-by-point manner, the DLP technique simultaneously exposes the entire layer via a selective masking approach. Since an entire layer is polymerized in a single exposure step, the build time is significantly reduced compared to point-by-point using a laser source. In the top-down DLP system used in this study (BMF S130, Boston Micro Fabrication, Maynard, MA, USA), polymerization occurs in a resin bath in which the light source is projected from above onto the liquid resin surface. This configuration minimizes detachment forces during layer formation and enables a higher printing resolution by maintaining a uniform resin surface [2,26].

Several recent studies have employed DLP to fabricate MNs, providing valuable insights into the morphological fidelity and mechanical performance of the MNs. However, most of these efforts focus on specific geometries or isolated process parameters rather than conducting systematic comparisons across multiple designs under standardized conditions. For example, Mathew et al. demonstrated that the print angle markedly affects the dimensional accuracy of hollow MN arrays, with mechanical strength assessed via compression and bending under different curing protocols [27]. Likewise, Tabriz et al. compared conical and pyramidal geometries with varying height-to-aspect ratios (1:1 to 3:1), showing that MN geometry strongly influences the piercing force, penetration depth, and insertion profile [28]. In another report, hollow MNs were evaluated using μCT, morphological imaging, and insertion testing, but only for a limited set of geometries [29]. Moreover, Razzaghi et al. revealed that variations in tilt angle or tip design can substantially reduce MN insertion forces, particularly when comparing solid and hollow MN architectures [30]. Despite these advances, there remains a need for a direct and comprehensive comparative study examining how different geometric designs (e.g., conical, pyramidal, arrowhead MN designs) affect both the morphological and mechanical properties of DLP-fabricated MNs. This gap is of a critical nature since MN geometry is a primary determinant of MN performance, directly affecting fracture resistance, insertion efficiency, and drug-loading capacity. To address this shortcoming, the present study systematically evaluates and compares MNs of multiple geometries fabricated under identical DLP conditions, thereby establishing design–property relationships that are essential for the rational development of transdermal drug delivery systems.

Herein, we present the fabrication of MNs with arrowhead, pyramid, and turret designs with three different height values (600 µm, 800 µm, and 1000 µm) using the DLP 3D printing method. The morphological and mechanical properties of 3D-printed MNs with different sizes and geometric designs were investigated, and the effects of these properties on transdermal drug delivery applications were evaluated. Within the scope of the study, MN designs were comparatively analyzed, and geometry-dependent structural and mechanical differences were revealed. The findings are intended to contribute to the optimization of DLP-based MN production and increase the efficiency of transdermal drug delivery via MNs.

## 2. Materials and Methods

### 2.1. Design and Fabrication of MN Arrays

**Figure 1:**
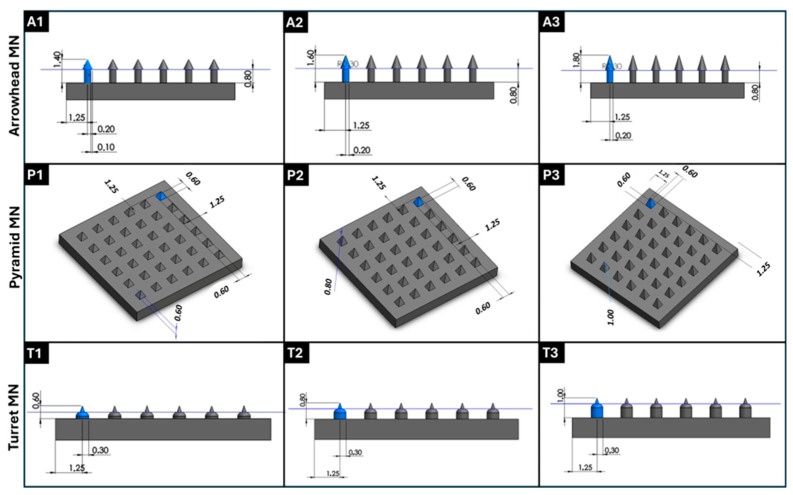
Three-dimensional designs of MN arrays with various geometries and dimensions were generated using SolidWorks 2020 software. (**A1**–**A3**) Arrowhead MN structures with various heights (600, 800, and 1000 µm) and widths of 600 μm; (**P1**–**P3**) top-view and perspective representations of pyramid MN arrays with different heights (600, 800, and 1000 µm); (**T1**–**T3**) turret MN geometries with height variations (600, 800, and 1000 µm). All designs were digitally modeled before DLP 3D printing for structural optimization and fabrication planning. Geometric parameters such as base diameter, height, and inter-needle spacing were adjusted accordingly.

Computer-aided design (CAD) files of 6 × 6 MN arrays featuring three distinct designs, specifically arrowhead, pyramid, and turret designs, each with three different dimensions, were created with SolidWorks 2020 software (Dassault Systèmes SE, Vélizy-Villacoublay, France). Arrowhead, pyramid, and turret MNs were designed with heights of 600, 800, and 1000 μm, which were selected to encompass the physiologically relevant range for skin penetration. These height values are sufficient to traverse the stratum corneum layer (10–20 μm) and viable epidermis layer (~50–100 μm) of the skin; the height values enable insertion into the upper dermis (~200–300 μm) for drug delivery, without reaching pain-inducing nerve endings or larger blood vessels [31]. All of the MN designs exhibited a base width of 600 μm (for the arrowhead MNs, this value refers to the MN tip base, not the MN shaft base), and were mounted on a 10 mm × 10 mm × 1 mm substrate (Figure 1). The MN design files were then sliced into layers using the BMF Slicer software (version 1.2) to prepare G-code compatible with the 3D printing instrument. A Model S130 digital light processing (DLP)-based 3D printer (Boston Micro Fabrication, BMF, Maynard, MA, USA) with a resolution of 2 μm in the XY plane and 5–20 μm in the Z axis was used to fabricate the MNs; this 3D printer contains a light source with a wavelength of 405 nm. The layer thickness (Z axis) was maintained at a value of 5 μm, the exposure time was maintained at a value of 1 s per layer, and the initial bottom exposure was maintained at a value of 3 s to ensure proper adhesion; a light intensity of 60 mW/cm^2^ was used in the study. The MN arrays were fabricated using a yellow-colored biocompatible photoreactive resin (BIO-Y-resin, BMF). Following the 3D printing process, the arrays were rinsed in isopropyl alcohol (IPA, 99.9% *v*/*v*) for 5 min to remove unpolymerized resin, and subsequently post-cured under a 405 nm lamp (Formlabs Inc., Somerville, MA, USA) at 45 °C for 10 min.

### 2.2. Morphological Characterization

The morphology of the 3D-printed arrowhead, pyramid, and turret MN arrays was evaluated in terms of height, base diameter, and surface roughness. A VK-X250 3D confocal laser scanning microscope (Keyence, Tokyo, Japan) was utilized to obtain quantitative topographical data. Measurements were obtained by positioning the MN array on its base and capturing a top-down 3D profilometry scan; the MN parameters were analyzed using Keyence MultiFile Analyzer software (version is 2.1.2.17) according to the standard surface analysis protocols of the instrument (ISO 25178 [32] for areal surface texture). Further analysis of the shape and morphological characteristics of the MN arrays was performed using a SU3900 variable pressure scanning electron microscope (VPSEM, Hitachi, Tokyo, Japan), which was equipped with a solid-state backscattered electron detector and operated using an accelerating voltage range of 0.3–30 kV. The SEM images were evaluated qualitatively in terms of geometry fidelity, tip definition, and surface integrity.

### 2.3. Mechanical Testing

Mechanical tests were undertaken to understand the fracture strength of the 3D-printed MN arrays using an EZ-LX mechanical testing machine (Shimadzu, Kyoto, Japan) that was equipped with a 500 N load cell in compression mode. The MN arrays were positioned on the flat surface of the steel base plate, and an axial force was applied perpendicularly to the axis of the MNs using a constant speed of 1 mm/min until fracture occurred. All tests were undertaken in triplicate (n = 3). This procedure followed previously reported MN compression testing protocols that are described in the literature [26,33,34,35]. The procedure is consistent with international recommendations for small-scale mechanical testing; it should be noted that no formal ISO/ASTM standard specific to MNs currently exists.

Engineering stress (σ, MPa) was calculated using the equation σ = F/A_0_, in which F is the applied force (N) and A_0_ is the cross-sectional area (mm^2^). For MN arrays, A_0_ was defined as the total footprint area of the array, which corresponds to the number of MNs multiplied by the base area of a single MN. Engineering strain (ε, %) was determined as ε = (ΔL/L_0_) × 100, in which ΔL is the change in MN height (mm) and L_0_ is the initial height (mm). All of the stress values were reported in MPa (1 N/mm^2^ = 1 MPa).

## 3. Results and Discussion

### 3.1. Profilometry Analysis

The geometry of the MN array is known to directly influence skin penetration, drug release mechanism, mechanical durability, patient comfort, and biological interactions [36]. Moreover, MNs designed with varying heights may be beneficial for regulating the penetration depth in the skin and adjusting the available volume for drug loading [11,37]. In this study, arrowhead (600 µm (A1), 800 µm (A2), and 1000 µm (A3)), pyramid (600 µm (P1), 800 µm (P2), and 1000 µm (P3)), and turret (600 µm (T1), 800 µm (T2), and 1000 µm (T3)) MN arrays designed in three different dimensions were successfully fabricated using the DLP 3D printing method.

**Figure 2:**
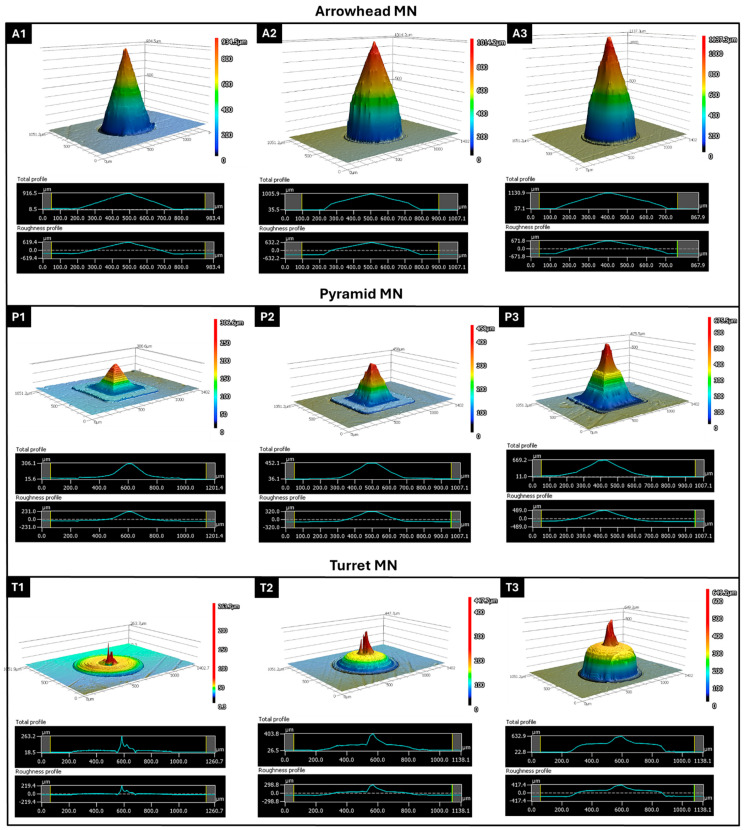
Three-dimensional confocal profilometry images and corresponding height/roughness profiles of 3D-printed MNs: arrowhead (**A1**–**A3**), pyramid (**P1**–**P3**), and turret (**T1**–**T3**) designs at 600, 800, and 1000 µm target heights. Height profiles show tip sharpness and surface integrity; roughness plots indicate surface variations (Ra, µm).

3D confocal laser scanning profilometry was performed on arrowhead (A1–A3), pyramid (P1–P3), and turret (T1–T3) designs to evaluate the surface topography and dimensional accuracy of the DLP-printed MNs (Figure 2). The results revealed significant differences in height profiles, base curvature, and surface roughness based on the geometry and design parameters. The arrowhead MNs (A1–A3) displayed a sharp conical morphology with smooth gradient transitions from the base to the tip; these images demonstrated that the DLP printing process achieved a high degree of vertical resolution fidelity. In the 3D reconstructions, however, the shaft region appeared truncated, an artifact stemming from the limitations of top-down confocal scanning in resolving steep, narrow sidewalls, where light scattering and signal attenuation hinder accurate profiling. This limitation was addressed by complementary SEM imaging, which confirmed the intact shaft morphology and overall fidelity of the printed arrowhead design. Confocal microscopy further showed that MN height increased progressively from A1 to A3, reaching ~1000 µm in A3, in line with the CAD specifications. A modest increase in surface roughness was also observed with increasing height (Ra: A1 ≈ 620 µm, A3 ≈ 672 µm). This trend can be explained by the cumulative effect of layer stacking during the vertical build process, since the printing layer thickness was set to 5 µm. At taller MN heights, a greater number of layers are required to complete the structure, which amplifies the visibility of interlayer interfaces and contributes to slightly higher surface roughness values. Nevertheless, the arrowhead constructs maintained a symmetrical profile, with well-defined tip geometries; these features are expected to support effective skin penetration while minimizing pain. Pyramidal MNs (P1–P3) presented a more structured height profile with flat, sloping edges and a prominent pyramidal apex. The measured heights of P1–P3 followed the expected increasing trend but deviated quantitatively from the CAD specifications (e.g., for P3, the value in the design is 1000 µm and the measured value is 660 µm). This discrepancy was most pronounced along the Z axis, reflecting cumulative shrinkage and voxel distortion during the vertical build process. In the XY plane, the overall base dimensions and pyramidal footprint were largely preserved, although slight rounding of the edges was noted; this result may be attributed to voxel bleeding and optical scattering at the feature boundaries [22,38]. The surface roughness across the pyramid group (Ra: ~230–490 µm) was slightly lower than that of the arrowhead group, particularly in P1. This lower roughness indicates more uniform layer deposition and a more stable print, especially in the lower height designs. In contrast, taller or more complex structures exhibited higher roughness values, which may lead to localized layer inconsistencies that affect overall print fidelity. Turret MNs with a wider base and truncated conical shape (T1–T3) exhibited a wider plateau region near the tip and smoother surface features. Profilometry scans showed that the height of the turret MNs was consistently lower than the design input (e.g., T3: ~610 µm vs. 1000 µm expected), suggesting slight underexposure or post-curing shrinkage in complex geometries [39]. Interestingly, the turret designs exhibited the lowest surface roughness values among all groups (Ra: ~220–418 µm), indicating smoother surfaces and more consistent layer fusion, especially at T2 and T3. These smoother surfaces may reduce the friction force during insertion and support long-term use in transdermal applications.

### 3.2. Morphological Analysis

**Figure 3:**
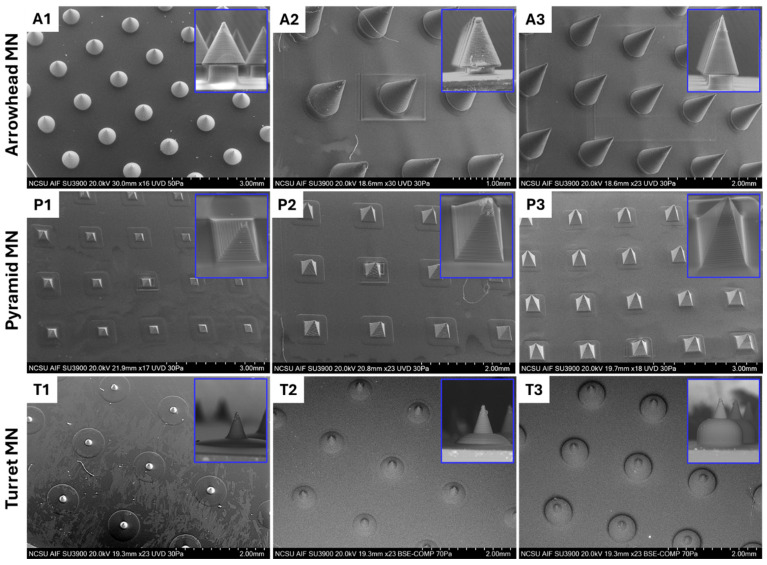
SEM images of 3D-printed arrowhead (**A1**–**A3**), pyramid (**P1**–**P3**), and turret (**T1**–**T3**) MN arrays designed at heights of 600, 800, and 1000 μm, respectively.

SEM images of 3D-printed arrowhead (A1–A3), pyramid (P1–P3), and turret (T1–T3) MN arrays are presented in Figure 3, along with a magnified image of the single MNs. The SEM images revealed that the MNs were fabricated without bending or fracture, and each form showed distinct structural differences. It should be noted that all of the MNs maintained their spacing consistently, and that each printed structure exhibited uniform and reproducible layers. The arrowhead (A1–A3) MN images showed that the component MNs possess uniform features. In magnified images, MNs were found to have sharp tips, and the aspect ratio was consistent with the computer-aided design model. In terms of dimension, the A1 MN array is shorter and may be suitable for drug delivery to upper layers of the skin; the A3 MN array may be more effective in applications requiring drug delivery to deeper skin layers. The A2 MN array is intermediate in length; since it has a shorter length than the A3 MN array, it may cause less pain and, as such, may have a broader range of applications [37]. Overall, arrowhead MNs can enhance the consistency of MN penetration through resisting the elastic structure of the skin, allowing the MNs to remain embedded at maximum penetration depths [9]. The images of the pyramid (P1–P3) MN arrays indicate a successful fabrication process, including uniform features in the component structures and the integration of the component structures into the base surface. In high magnification images, stair-stepping was observed in P1, P2, and P3 MN structures, which was associated with a jagged MN sidewall. Such jagged surfaces may lead to stress concentration points, thereby reducing the overall fracture resistance and mechanical integrity of the MNs during insertion. Although these imperfections do not hinder successful fabrication, their presence highlights the importance of optimizing printing parameters such as layer thickness to minimize sidewall roughness and ensure reliable performance [40]. In addition to layer thickness, several other parameters may be optimized to minimize surface defects, such as laser power, scanning speed, exposure time, and resin viscosity. These factors influence the polymerization kinetics and voxel overlap during the 3D printing process, which in turn determine the smoothness and uniformity of the MN sidewalls [41]. Pyramid MN arrays can increase skin penetration capacity by offering a more rigid form than arrowhead MNs. P3 MNs, in particular, can show high efficiency in terms of tissue penetration due to their sharp edges. However, the surface roughness of these MNs can affect drug capacity and interactions with the skin. Turret MNs, featuring sharp tips and a wide base, can easily puncture the skin while offering enhanced mechanical strength [9]. SEM analyses of turret (T1–T3) MN arrays indicate that these MNs exhibit a smooth surface; however, their penetration capacity may be lower compared to other designs. Compared to the T1 and T2 MN arrays, the T3 MN array most accurately exhibited the turret morphology and is associated with lower skin penetration, which may be advantageous for applications that are associated with a low pain threshold.

### 3.3. Mechanical Performance Evaluation

**Figure 4:**
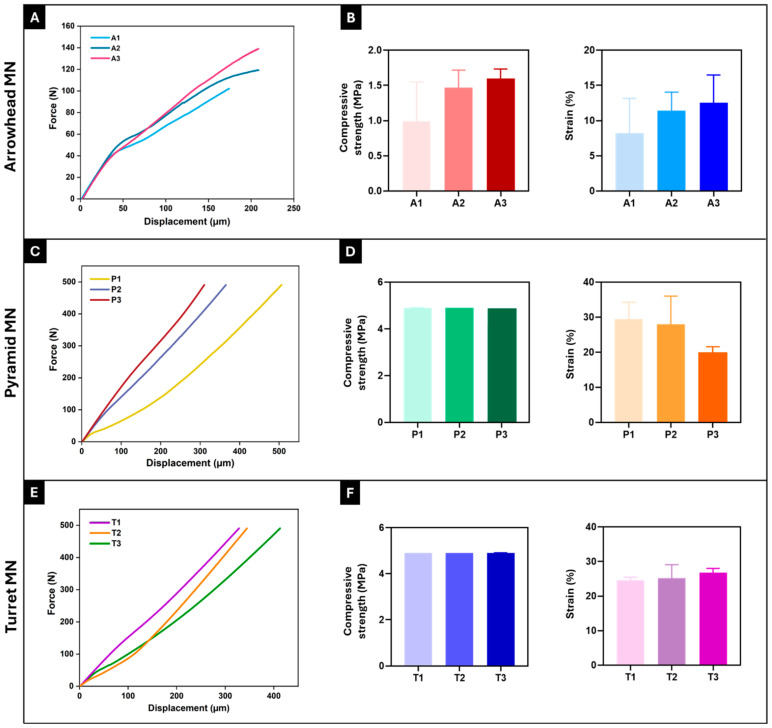
Force-displacement curves and compressive strength and strain (%) values of 3D-printed arrowhead (**A**,**B**), pyramid (**C**,**D**), and turret (**E**,**F**) MN arrays, respectively. Curves (**A**,**C**,**E**) represent the average force-displacement data obtained from three independent tests (n = 3) for each design (A1–A3, P1–P3, T1–T3). Error bars represent the standard deviations (SDs) of measurements performed on at least three samples (n = 3).

To effectively penetrate the skin barrier and deliver the drug payload, the mechanical properties and insertion capability of MNs are important parameters [28]. The mechanical performance of 3D-printed arrowhead (A1–A3), pyramid (P1–P3), and turret (T1–T3) MN arrays was evaluated via compression testing. The force-displacement curves for each MN design were averaged from three replicate tests (n = 3), and these average curves are presented in Figure 4A,C,E. As shown in Figure 4A,C,E, the force-displacement curves of the 3D-printed MN arrays remained continuous at a given displacement, with no signs of fracture or rupture, indicating the toughness of MNs. An examination of the mechanical testing results for arrowhead MNs revealed a general increase in force values with increasing MN height. The A3 MN design reached the highest force value (140 N) at 208.5 µm displacement compared to A1 and A2 (Figure 4A); it also exhibited the highest compressive strength (1.6 ± 0.1 MPa) and strain (12.5 ± 3.2%) values (Figure 4B). Analysis of the force-displacement curves of the pyramid MNs showed no significant difference in the force values (~491 N) applied to the P1, P2, and P3 MNs. However, the lowest displacement was noted in the P3 MN design, with a value of 272 µm (Figure 4C). The sharp-edged structure of pyramid geometry is known to enhance the load-carrying capacity, thereby improving mechanical strength [15,36]. It was observed that the strength increased significantly as the MN height increased in the pyramid designs. The compressive strength (4.9 MPa) and strain (20.1 ± 1.7%) values of the P3 MN design also support this result (Figure 4D). These results are consistent with similar studies reported in the literature [42,43]. Evaluation of the force-displacement curves of the turret MNs (T1–T3) demonstrated a more linear force increase compared to the other two designs, along with the most stable mechanical performance. Although an increase in MN height did not affect the force applied (~491 N) to the MNs, the T1 MN array exhibited the lowest displacement (328.5 µm) (Figure 4E). The compressive strength (4.9 MPa) and strain (24.5 ± 0.7%) values of the T1 design further validated this finding (Figure 4F). An increase in MN height led to a slight reduction in the mechanical strength of the turret MNs. In summary, the arrowhead design exhibited a lower load-carrying capacity than the pyramid and turret designs. This result suggests that the arrowhead geometry may be more susceptible to bending and torsional forces. The P3 MN array exhibits the highest mechanical strength among the designs, whereas the turret MNs provide more stable performance. However, it is important to consider that higher mechanical strength may increase the risk of pain and tissue damage during penetration [36]. These findings align with previous studies, which have shown that the mechanical stability of MNs is influenced not only by the material type but also by the geometry [10,11,44].

To understand the practical applicability of the fabricated MN arrays, it is essential to compare their mechanical strength with the force required for human skin penetration. The measured failure forces of all of the designs in this study (ranging from ~50 N to >400 N per array, Figure 4) substantially exceed the typical insertion forces reported for MNs, which are approximately 0.1–3 N per MN [26,45,46]. This considerable safety margin suggests that the fabricated designs, particularly the pyramid and turret geometries, possess sufficient robustness to penetrate the skin without fracture. Nevertheless, it is important to note that mechanical strength alone does not ensure successful insertion or superior performance. Additional factors, such as insertion efficiency, safety, and patient comfort, must also be considered and will require systematic investigation in future studies [8]. Taken together, these findings highlight the promise of 3D-printed MN designs as viable candidates for transdermal drug delivery applications.

## 4. Conclusions

This study demonstrated that DLP-based 3D printing is an effective technique for fabricating MN arrays in arrowhead (A1–A3), pyramid (P1–P3), and turret (T1–T3) designs with three different heights (600 µm, 800 µm, and 1000 µm). The printed MNs were characterized for their morphological and mechanical properties, revealing that mechanical performance varies significantly based on the geometric design. According to the results, arrowhead MNs had a lower load-bearing capacity and were more susceptible to bending, whereas the pyramid and turret designs exhibited greater mechanical stability and improved penetration ability. Among the MN designs, the pyramid design (P3) demonstrated the highest mechanical strength, while turret MNs maintained more consistent performance despite having a reduced penetration capacity. These findings emphasize the importance of geometric design for determining the mechanical stability and performance of MNs for transdermal drug delivery applications, with implications for optimizing MN design to balance penetration depth and mechanical stability.

## Data Availability

The original contributions presented in this study are included in the article. Further inquiries can be directed to the corresponding author.
